# Successful treatment of a patient with cutaneous co-infection caused by *Mucor irregularis* and *Klebsiella pneumoniae*^☆^^☆☆^

**DOI:** 10.1016/j.abd.2020.03.004

**Published:** 2020-06-16

**Authors:** Siping Zhang, Kunju Zhu, Chi Zhang

**Affiliations:** a Department of Dermatology and Venereology, First Affiliated Hospital, University of Science and Technology of China, Hefei, Anhui, China; b Department Institute of Clinical Medicine Research, First Affiliated Hospital, Jinan University, Guangzhou, Guangdong, China

**Keywords:** Amphotericin B, *Klebsiella pneumoniae*, Mucormycosis

## Abstract

The authors report a rare case of primary cutaneous mucormycosis caused by *Mucor irregularis* and cutaneous *Klebsiella pneumoniae* infections in a 67-year-old Chinese woman. After the administration of liposomal amphotericin B combined with cefoperazone/sulbactam sodium, the patient recovered. Invasive fungal infection combined with cutaneous bacterial infection should receive attention.

## Introduction

Mucormycosis is a rare, invasive fungal infection with exceedingly high mortality and few therapeutic options; it frequently occurs in immunocompromised patients. Many species belonging to the order Mucorales can cause mucormycosis, but mucormycosis caused by *Mucor irregularis* (formerly *Rhizomucor variabilis*) is extremely rare. This report describes a case of slowly progressive *Mucor irregularis* infection on the face accompanied by cutaneous *Klebsiella pneumoniae* infection.

## Case report

**Figure 1:**
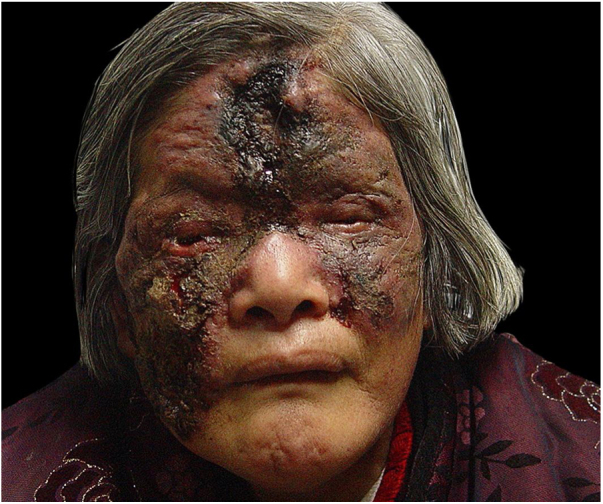
Before treatment.

A 67-year-old female presented with a nine-year history of a red lesion on the face after a clear left eyelid puncture wound. She had fever, dehydration, vomiting, and body pain in the first interview. Cutaneous examination showed an ulcer with a purulent central yellow secretion, and a hard, dry, black crust on the forehead and right cheek surrounded by an inflammatory ring. There was an irregular, confluent, infiltrated, light-red plaque with a clear boundary involving her nose, left cheeks, and eyelids. A massive brownish-red necrosis scab covered most parts of the lesions Lesions were tender and pruriginous. Submandibular and neck lymph nodes could be palpated (Fig. 1).

**Figure 2:**
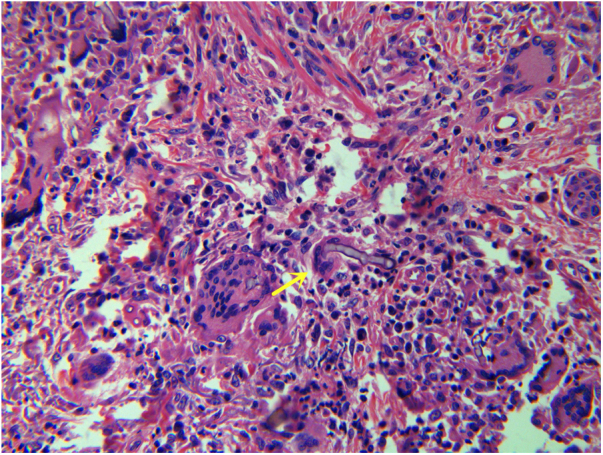
Ribbon-like, nonseptate hyphae characteristic of a mucoralean fungus in multinucleated giant cell (yellow arrow; Hematoxylin & eosin, ×400).

**Figure 3:**
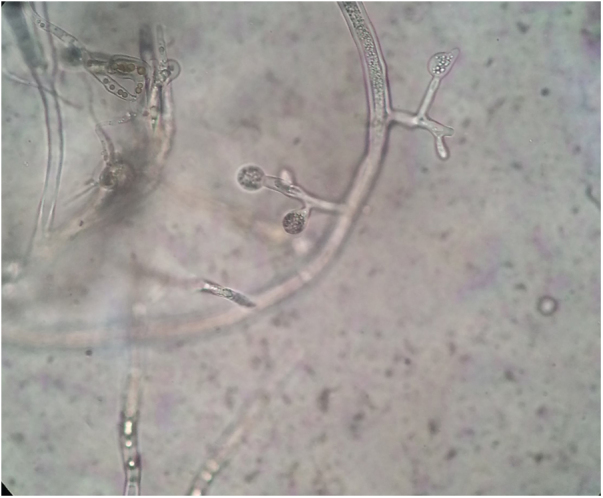
Slide culture revealed spherical sporangia, long and hyaline hyphae, and rhizoids with no distinguishable stolons (×400).

Complete blood cell count showed a mild anemia. Plain computed tomography (CT) scan and enhancement showed a soft tissue shadow without a distinctive border. The craniofacial bone was intact. Histopathological examination of tissue biopsy with hematoxylin and eosin revealed ribbon-like, nonseptate hyphae characteristic of a mucoralean fungus in multinucleated giant cell (Fig. 2). The bacterial culture isolate harbored the wild-type resistant phenotype of *K. pneumoniae*. Microscopic examination of the slide culture revealed spherical sporangia, long and hyaline hyphae, and rhizoids with no distinguished stolons (Fig. 3). Biomolecular identifications were confirmed by internal transcribed spacer (ITS) region nucleotide sequencing (primer sequence: ITS1 5′-TCCGTAGGTGAACCTGCGG-3′, ITS4 5′-TCCTCCGCTTATTGATATGC-3′) and sequence similarity search using BLAST in the National Center for Biotechnology Information database (NCBI). A BLAST search of the NCBI GenBank database showed that the isolated pathogen had 99% homology with that of *M. irregularis* (GenBank accession No. EF583637).

**Figure 4:**
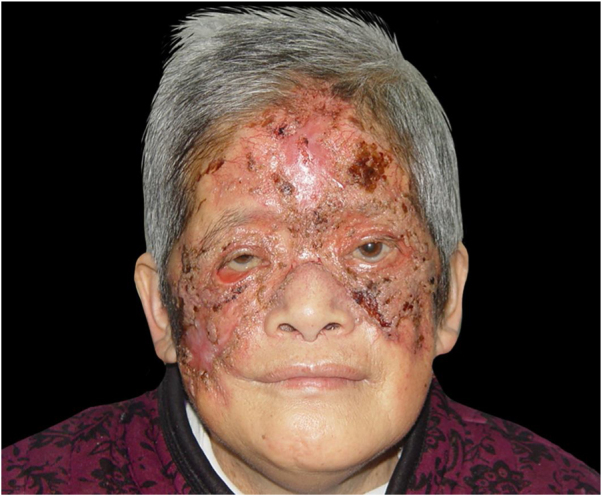
After treatment, the superficial ulcer became drier with the formation of fresh granulation tissue, and the necrotic crust fell off, while purulent secretions disappeared.

The patient was treated with EV amphotericin B (AmB) combined with cefoperazone/sulbactam sodium according to a drug susceptibility test. AmB was administered gradually from a daily dose of 1 mg to a maximum daily dose of 40 mg. After one week, the superficial ulcer became drier with the formation of fresh granulation tissue, and the necrotic crust fell off, while purulent secretions disappeared. However, side effects gradually appeared: high fever, runny nose, muscular soreness, headache, vomiting, and unbearably severe hypokalemia and hypoproteinemia. Renal function started to slightly deteriorate. Thus, AmB was replaced by liposomal AmB, which was more effective and presented fewer side effects in the treatment of invasive fungal infections.^1^ With the side effects relieved, the lesion recovered gradually (Fig. 4). Fungal examination of the skin lesion yielded negative results. When the total dose reached 1.5 g, AmB treatment was discontinued and was replaced by oral itraconazole (400 mg per day) for two months. No recurrence was observed during the three-month follow-up period after discontinuing the oral itraconazole treatment.

## Discussion

*Mucor irregularis* infection occurs mainly in patients with predisposing factors such as diabetes, malignancy, solid organ transplantation, and trauma. However, most of the patients reported with mucormycosis due to *M. irregularis* did not present immunodeficiency.^2^ Our patient reported a local trauma with a plant and did not present immunodeficiency according to blood examination. Systematic application of glucocorticoid causing the immunosuppression of the patient shortly before her visit to the clinic may have been the trigger of aggravation of the disease. The clinical manifestations of and subsequent mortality due to cutaneous mucormycosis are dependent on the mode of acquisition and the host's immune status. This fact highlights the importance of assessing the history of immunosuppressive agents.

*K. pneumoniae* is a well-known Gram-negative pyogenic pathogen, which is one of the most common community-acquired pathogens, causing eye, brain, lung, liver, and genitourinary infections. Occasionally, *K. pneumoniae* can give rise to complicated skin and soft-tissue infections, which have been reported to present as necrotizing fasciitis and soft-tissue infection, or as deep abscess and purpuric rash. Most of the reported cases are usually combined with complicated skin and soft-tissue infections. Cutaneous mixed infection with *M. irregularis* and *K. pneumoniae* in the skin is an extremely rare event.

Treatment of invasive fungal infections is complicated because the drug target sites of eukaryotic pathogens closely resemble those of the human host, which limits therapeutic options.^3^ Primary cutaneous mucormycosis results from direct inoculation of fungal spores into skin, whose resulting lesions may mimic pyoderma gangrenosum, bacterial synergistic gangrene, or other infections produced by bacteria or fungi. The highly variable clinical presentation, non-specific findings of infection, and the higher prevalence of other infectious conditions are responsible for delay in cutaneous mucormycosis diagnosis. Our patient was diagnosed 10 years after the onset of the skin lesions.

The management in such cases is often a challenging task, as the delayed diagnosis poses a major hindrance for an early treatment. Based on the available evidence, AmB is the appropriate empirical antifungal for invasive mucormycosis, and possesses the largest spectrum of antifungal activity and susceptibility. Utilizing liposomal formulations or other strategies to reduce nephrotoxicity is prudent.^3^ Compared with conventional AmB, lipid formulations of AmB are now the treatment of choice and should be administered intravenously at a minimum of 3–5 mg/kg/day.^4^ Nonetheless, its high cost is a constraint in developing countries, and patients usually do not comply with the full course of treatment. Although *M. irregularis* is less susceptible to fluconazole and itraconazole, the successful treatment of cutaneous mucormycosis with these antifungals has been reported in the literature.^5^ According to bacteria culture results, our patient received oral itraconazole and intravenous liposomal AmB, as well as cefoperazone/sulbactam sodium.

## Financial support

None declared.

## Authors’ contributions

Siping Zhang: Approval of final version of the manuscript; conception and planning of the study; drafting and editing of the manuscript; collection, analysis, and interpretation of data; participation in study design; intellectual participation in the propaedeutic and/or therapeutic conduct of the studied cases; critical review of the literature.

Kunju Zhu: Approval of final version of the manuscript; drafting and editing of the manuscript; participation in study design; critical review of the literature; critical review of the manuscript.

Chi Zhang: Approval of final version of the manuscript; drafting and editing of the manuscript; collection, analysis, and interpretation of data; participation in study design; intellectual participation in the propaedeutic and/or therapeutic conduct of the studied cases; critical review of the literature; critical review of the manuscript.

## Conflicts of interest

None declared.

## References

[1] Moen M.D., Lyseng-Williamson K.A., Scott L.J. (2009). Liposomal amphotericin B: a review of its use as empirical therapy in febrile neutropenia and in the treatment of invasive fungal infections. Drugs.

[2] Xia X.J., Shen H., Liu Z.H. (2015). Primary cutaneous mucormycosis caused by *Mucor irregularis*. Clin Exp Dermatol.

[3] Riley T.T., Muzny C.A., Swiatlo E., Legendre D.P. (2016). Breaking the mold: a review of mucormycosis and current pharmacological treatment options. Ann Pharmacother.

[4] Petrikkos G.L. (2009). Lipid formulations of amphotericin B as first-line treatment of zygomycosis. Clin Microbiol Infect.

[5] Zhao Y., Zhang Q., Li L., Zhu J., Kang K., Chen L. (2009). Primary cutaneous mucormycosis caused by *Rhizomucor variabilis* in an immunocompetent patient. Mycopathologia.

